# Induction of beta defensin 2 by NTHi requires TLR2 mediated MyD88 and IRAK-TRAF6-p38MAPK signaling pathway in human middle ear epithelial cells

**DOI:** 10.1186/1471-2334-8-87

**Published:** 2008-06-25

**Authors:** Haa-Yung Lee, Tamotsu Takeshita, Jun Shimada, Arsen Akopyan, Jeong-Im Woo, Huiqi Pan, Sung K Moon, Ali Andalibi, Rae-Kil Park, Sung-Ho Kang, Shin-Seok Kang, Robert Gellibolian, David J Lim

**Affiliations:** 1 The Gonda Department of Cell and Molecular Biology, House Ear Institute, Los Angeles, CA, USA; 2 Department of Otorhinolaryngology, Hamamatsu University School of Medicine, Hamamatsu, Japan; 3 Department of Otolaryngology and Head & Neck Surgery, Wakayama Medical University, Wakayama, Japan; 4 Department of Microbiology, Wonkwang University School of Medicine, Iksan, South Korea; 5 Department of Otolaryngology, Konkuk University School of Medicine, Chungju, South Korea; 6 Department of Otolaryngology, University of Southern California, Los Angeles, CA, USA; 7 Department of Cell and Neurobiology, University of Southern California, Los Angeles, CA, USA

## Abstract

**Background:**

All mucosal epithelia, including those of the tubotympanium, are secreting a variety of antimicrobial innate immune molecules (AIIMs). In our previous study, we showed the bactericidal/bacteriostatic functions of AIIMs against various otitis media pathogens. Among the AIIMs, human β-defensin 2 is the most potent molecule and is inducible by exposure to inflammatory stimuli such as bacterial components or proinflammatory cytokines. Even though the β-defensin 2 is an important AIIM, the induction mechanism of this molecule has not been clearly established. We believe that this report is the first attempt to elucidate NTHi induced β-defensin expression in airway mucosa, which includes the middle ear.

**Methods:**

Monoclonal antibody blocking method was employed in monitoring the TLR-dependent NTHi response. Two gene knock down methods – dominant negative (DN) plasmid and small interfering RNA (siRNA) – were employed to detect and confirm the involvement of several key genes in the signaling cascade resulting from the NTHi stimulated β-defensin 2 expression in human middle ear epithelial cell (HMEEC-1). The student's *t*-test was used for the statistical analysis of the data.

**Results:**

The experimental results showed that the major NTHi-specific receptor in HMEEC-1 is the Toll-like receptor 2 (TLR2). Furthermore, recognition of NTHi component(s)/ligand(s) by TLR2, activated the Toll/IL-1 receptor (TIR)-MyD88-IRAK1-TRAF6-MKK3/6-p38 MAPK signal transduction pathway, ultimately leading to the induction of β-defensin 2.

**Conclusion:**

This study found that the induction of β-defensin 2 is highest in whole cell lysate (WCL) preparations of NTHi, suggesting that the ligand(s) responsible for this up-regulation may be soluble macromolecule(s). We also found that this induction takes place through the TLR2 dependent MyD88-IRAK1-TRAF6-p38 MAPK pathway, with the primary response occurring within the first hour of stimulation. In combination with our previous studies showing that IL-1α-induced β-defensin 2 expression takes place through a MyD88-independent Raf-MEK1/2-ERK MAPK pathway, we found that both signaling cascades act synergistically to up-regulate β-defensin 2 levels. We propose that this confers an essential evolutionary advantage to the cells in coping with infections and may serve to amplify the innate immune response through paracrine signaling.

## Background

The respiratory mucosal epithelia, including the middle ear mucosa, are directly exposed to the environment and serve as an effective first line of defense against a variety of potentially pathogenic microorganisms. Second only to the common cold, otitis media (OM) is the most prevalent mucosal infectious disease affecting young children. OM results in 31 million annual visits to physicians' offices and is estimated to have a yearly cost exceeding $5 billion USD. Nontypeable *Haemophilus influenzae *(NTHi) [1,2] is one of the major OM pathogens and is also a causing agent for sinusitis and chronic obstructive pulmonary disease (COPD) [3]. In the past three decades, there has been a dramatic worldwide increase in antibiotic resistance in respiratory pathogens. There is, thus, an urgent need to develop new and innovative, non-antibiotic approaches to prevent and manage this disease [4,5]. The pathogenesis of OM is multi-factorial and it is believed that the antimicrobial innate immune molecules (AIIMs) [6-11] as well as pathogen recognition receptors such as the toll-like receptors (TLR) are playing roles in OM susceptibility [3,12,13].

Innate immune molecules such as lysozyme, lactoferrin, PLUNC (palate, lung and nasal epithelium clone) and defensins are produced by the mucosal epithelial cells and provide the host with continuous innate immunity against a variety of invading pathogens [14,15]. Among the AIIMs, the β-defensin family is one of the most potent innate immune molecules [16-18]. Some of the major roles defensins play in host defense are direct antimicrobial activity, facilitation and amplification of innate and adaptive immunity [19-22]. To date, multiple β-defensin genes from epithelial and epididymal cells have been identified [23-25]. Among them, four of epithelial β-defensins (HBD 1–4) have been characterized at the peptide level [23,24,26]. β-defensin 1 is expressed constitutively by a variety of cell types, while β-defensin 2 expression is highly up-regulated by exposure to inflammatory stimuli such as bacterial components or proinflammatory cytokines [20,23]. We have recently shown that both human β-defensin 1 and 2 (HBD-1 and -2) have bactericidal/bacteriostatic activity against NTHi [14]. Moreover, in a previous study, we demonstrated that IL-1α up-regulates the transcription of HBD-2 in human middle ear epithelial cells (HMEEC-1), through the Src dependent Raf-MEK1/2-ERK signaling pathway [27]. However, despite this common generality, the degree of HBD-2 induction was variable in different cell types in response to inflammatory signal [20,28]. In human skin keratinocytes, the *E. coli *LPS is a weak inducer of HBD-2 signaling, but the induction is greatly increased when monocyte-derived cells were used as intermediaries between LPS and the epidermal keratinocytes. This may be due to the facts that the amplified epidermal response to LPS is mediated through the IL-1 pathway which is the dominant inducer of HBD-2 [20,29,30]. It is possible that HBD-2 and IL-1 signal amplification is mediated through TLRs.

The innate immune system has been known to utilize the Toll-like receptors (TLRs) for recognition of pathogen-associated molecular patterns (PAMPs), thus activating the MAPK or the NF-kB-dependent cell signaling cascades, resulting in a rapid, full-blown proinflammatory response [19]. TLR proteins are a family of type I trans-membrane receptors consisting of an NH_2_-terminal extracellular leucine-rich repeat domain (LRR) and a COOH-terminal intracellular Toll/IL-1 receptor (TIR) homology domain [31,32]. The intracellular TIR domain is a region of 150 amino acids with three highly conserved regions [33]. This TIR domain plays an important role in mediating protein-protein interactions between TLR proteins and their downstream signal transduction components such as the MyD88 protein [13]. The MyD88 recruited via its TIR domain to the TLR receptor promotes association with the interleukin-1 receptor-associated kinase 1 (IRAK1) [13,34]. The phosphorylation of IRAK1 results in its dissociation from the complex and its interaction with the tumor necrosis factor receptor-associated factor 6 (TRAF6) [13,34]. Activation of the TLR2-MyD88-IRAK1-TRAF6 can lead to the activation of many downstream signaling pathways, including members of the mitogen-activated protein kinase (MAPK) family [19,35]. At least three MAPK families have been identified to date: extracellular signal-regulated kinase (ERK), c-Jun kinase (JNK/SAPK) and p38 MAPK [19]. Among the three possible MAPK pathways, we hypothesize that NTHi WCL-induced HBD-2 up-regulation in HMEEC-1 takes place through the p38 MAPK cascade. Furthermore, TIR-like motifs in receptors from various plant species have also been shown to confer disease resistance [33], indicating a evolutionarily conserved functional role for this domain across species. Even though the IL-1 receptor and TLR are composed of different extracellular receptors, similarities between their intracellular TIR domains leads them to exhibit similar immunological responses [32]. This fact leads us to hypothesize that the possible interaction of the two domains is important to the synergistic induction of HBD-2.

In this study we show that NTHi12 induced HBD-2 up-regulation mainly takes place through the TLR2-MyD88-IRAK1-TRAF6-MKK3/6-p38 MAPK pathway. This result may also explain the significant synergistic effects of NTHi and IL-1α co-stimulation in expression and regulation of HBD-2 [30].

## Methods

### Bacterial culture and preparation of whole cell lysate

The NTHi strain 12 used in this study is a clinical isolate and it has been well documented [14,36,37]. The preparation procedure was described in a previous paper [14]. Briefly, stocks of NTHi 12 (obtained from Dr. Steve Barenkamp, St. Louis University, School of Medicine) were maintained at -80°C. The bacteria were plated on chocolate agar and incubated overnight at 37°C in 5% CO_2_. A single colony was used to inoculate 10 ml of brain heart infusion (BHI, Becton Dickinson, Cockeysville, MD), supplemented with hemin (10 μg/ml, Sigma, St. Louis, MO) and nicotinamide adenine dinucleotide (NAD10 μg/ml, Sigma, St. Louis, MO), and allowed to grow overnight. The bacteria were collected at 5,000 × g for 10 min and washed three times in phosphate buffered saline (PBS). After sonication, the lysate was cleared by centrifugation at 10,000 × g for 10 min. The cleared whole cell lysate (WCL) was collected and stored at -80°C and the pellet (P) was resuspended in PBS. To compare variations of HBD-2 induction by different NTHi strains, we prepared WCL from NTHi 2019 and NTHi 9274 (kind gifts from Dr. Michael Apicella, The university of Iowa, Department of Microbiology) using the same method described above [38-40].

### Mammalian epithelial cell culture

The human middle ear epithelial cell line (HMEEC-1) used in this study was immortalized with the E6/E7 genes of human papilloma virus type 16 and has been used in number of cell signaling studies [27,30,41,42]. HMEEC-1 cells were maintained in a 1:1 mixture of Dulbecco's modified Eagle's medium (DMEM) (Life Technologies, Inc, Gaithersburg, MD) and Bronchial Epithelial Basal Medium (BEBM) (Clonetics, Walkersville, MD) supplemented with bovine pituitary extract (52 μg/ml), hydrocortisone (0.5 μg/ml), hEGF (0.5 ng/ml), epinephrine 0.5 (μg/ml), transferrin (10 μg/ml), insulin (5 μg/ml), triiodothyronine (6.5 ng/ml), retinoic acid (0.1 ng/ml), gentamycin (50 μg/ml) and amphotericin-B (50 ng/ml). All cells were cultured in a humidified atmosphere of 5% CO2 and 95% air. A549 (human lung carcinoma) cell lines were cultured in DMEM supplemented with 5% fetal bovine serum (Life Technologies, Inc., Gaithersburg, MD).

### Blocking TLR2/TLR4 with monoclonal-Antibody

HMEEC-1 cells were cultured to 80% confluence and treated with 10 μg/ml of human TLR2 or TLR4 blocking antibodies (eBioscience, San Diego, CA) for 30 minutes at room temperature followed by stimulation with 5 μg/ml NTHi WCL for 4 hours. Isogenic antibody (IgG2a, k) was used as the control. All experiments were done in triplicates.

### Plasmid transfection and chemical inhibitors

HMEEC-1 cells were cultured in 12 well plates for 24 hours and then transiently transfected with appropriate dominant-negative mutant (DN) plasmids – hTLR2 DN, MyD88 DN, and TRAF6 DN (described previously [43,44]) and hTLR4 DN and IRAK1 DN (kindly provided by Dr. Jian-Dong Li) and pcDNA3.1 (Invitrogen, Carlsbad, CA) served as the control. All transient transfections were carried out in triplicate using a concentration of 1 μg/ml of plasmid and TransIT-LT1 reagent (Mirus, Madison, WI) following the manufacturer's instructions. 42 hours after transfection, 5 μg/ml of NTHi WCL was added to the cells for 4 hours. In all DN transfection experiments, a background vector was used as a negative control. For chemical signal blocking study, the HMEEC-1 cells were cultured for 2 days to 80% confluence. The cells were grown overnight in basic medium without supplement then pre-treated with 5 μM chemical inhibitors, SB203580, PD98059, U0126 (Calbiochem Inc., La Jolla, CA) or vehicle for 1 hour followed by treatment with NTHi WCL for 4 hours.

### Animal studies

10 week-old C57BL/6 male mice were used for studying the *in vivo *kinetic of NTHi induced mBD-2 expression. All aspects of animal handling were performed according to approved HEI IACUC protocols. The mice were transtympanically inoculated with 10 μl of the NTHi WCL after being anesthetized with Ketamine (5 mg/100 g). 1× PBS solution (10 μl) was used as the negative control. At 0, 6, 9, 12 and 24 hours post-inoculation, the middle ear mucosal RNA of three mice were harvested by irrigation of the bulla with three, 3.5 μl volume of Trizol (Invitrogen). The mice were sacrificed with CO_2 _and then decapitated. A small incision (1 cm) was made in the retroauricular area and the cortical bone of the bulla was exposed after dissection. A 2 mm × 2 mm sized hole was made using a sharp scalpel followed by irrigation of the bulla with Trizol. The total Trizol volume was then increased to 200 μl and RNA was precipitated using the manufacturer's instructions. The middle ear mucosa was inflamed in all NTHi-treated mice, but effusion was not seen in all groups. No sepsis or death occurred as a result of the experimental treatment.

To compare mouse β-defensin 2 (mBD-2) expression in TLR2 KO and wild type animals, the right ear was treated with NTHi WCL while the left ear served as a PBS treated control. The middle ear mucosal RNA was collected at 4 hours post-inoculation and analyzed by real-time quantitative PCR.

### RNA extraction and real-time quantitative RT-PCR analysis

RNA from cell lines or middle ear mucosa was extracted using Trizol (Invitrogen, Carlsbad, CA) and cDNA was synthesized using SuperScript II RNase H^- ^reverse transcriptase according to the manufacturer's protocol (Life Technologies, Inc, Gaithersburg, MD). Applied Biosystems' Assays-on-Demand primer and probe sets were then used to perform real-time quantitative PCR on each sample. In each case, the Cyclophilin gene served as the internal standard. The assays used were as follows: 1) Defb2 (mouse β-defensin 2), ABI assay number Mm00657074_m1(NM_010030.1); 2) DEFB4 (human β-defensin 2/human β-defensin 4), ABI assay number Hs00823638_m1 (NM_004942); 3) PPID (human cyclophilin D), ABI assay number Hs00234593_m1 (NM_005038); 4) Ppid (mouse cyclophilin D) ABI assay number Mm00835365_g1 (NM_026352). The relative quantity (CT treshhold method) of β-defensin 2 mRNA was quantitated.

### Western blot analysis and kinase assays

Cells were lysed using a buffer solution containing 25 mM TrisCl, 1 mM EDTA, 5 mM MgCl 2, 1 mM DTT, 100 mM NaCl, 10% glycerol, 1% Triton ×-100, 1 mM PMSF, and 5 μg/ml each of leupeptin, aprotinin, and pepstatin. The homogenate was centrifuged at 13,000 rpm for 10 min and the supernatant collected. Protein concentration was measured with the BCA protein assay kit (Bio-Rad, Inc., Richmond, CA). Cell lysates containing 50 μg of protein were boiled for 5 min in reducing SDS-PAGE sample buffer (0.125 Tris-HCl, pH 6.8, 4% SDS, 20% glycerol, 10% β-mercaptoethanol, and 0.2% bromophenol), and run on SDS-PAGE. The bands were transferred to a 0.2-μm pore nitrocellulose membrane (Whatman, Sanford, ME) in 20% methanol, 25 mM Tris, and 192 mM glycine, pH 8.3. The membrane was blocked with 5% milk, followed by exposure to the primary antibody (p38 α/β and phospho-p38 α/β, Cell Signaling Inc., Beverly, MA). A secondary antibody coupled to horseradish peroxidase (1:10,000; Amersham Biosciences) was used to bind to the primary antibody and be detected using enhanced chemiluminescence (NEN Life Science Products).

### siRNA Transfection and ELISA

Small interfering RNA (siRNA) was used for transient gene knock-down studies. All transient transfections were carried out in triplicate using *NeoFX *reagent (Ambion, Austin, TX) to final concentration of 10 nM following the manufacturer's instructions. The siRNAs used were as follows: 1) TLR2, siRNA #111285; 2) MyD88, siRNA #11601; 3) IRAK1, siRNA #138; 4) TRAF6, siRNA #107476; 5) MKK3, siRNA #1609; 6) MKK6, siRNA #1321; 7) p38, siRNA #1312 and siRNA #1 for the negative control. After 20 hours, the transfected cells were grown in fresh medium and cultured for another 48 hrs. The efficiency of gene knock down was evaluated by either RT-PCR or quantitative PCR. The cells were then exposed to NTHi WCL (5 μg/ml) for either 4 hours (gene expression study) or 24 hours (measuring protein). In the latter case, the culture supernatant was harvested and measured by ELISA (Phoenix Pharmaceuticals, Inc., Belmont, CA). For gene expression analysis, total RNA was isolated from the cells and used for real-time quantitative PCR.

### Statistics

All experiments were carried out in triplicate. Results are expressed as mean ± standard deviation. The student's t-test was used to measure significance.

## Results

### NTHi up-regulates β-defensin 2 expression in mucosal epithelial cells *in vitro *and *in vivo*

#### In vitro

**Figure 1:**
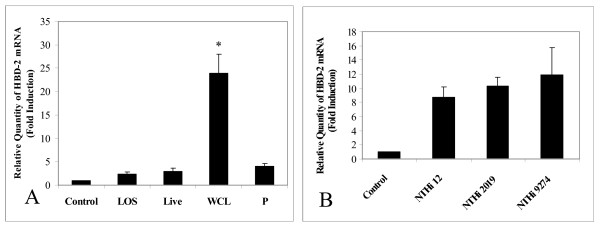
**Induction of β-defensin 2 (HBD-2) in human middle ear epithelial cells (HMEEC-1) using various NTHi preparations or NTHi variants.** With the exception of lipoolygosaccharide (LOS), all other NTHi preparations were derived from the same colony of NTHi strain 12. The LOS was purified from NTHi 12 and used at a concentration of 500 ng/ml. Live NTHi12 was treated using an multiplicity of infection (MOI) of 100 and NTHi WCL (whole cell lysate) and P (Pellet) were used at a concentration of 5 μg/ml. (A), The WCL treatment induced substantially higher expression of HBD-2 than other preparations. (B), Different strains of NTHi WCL were prepared and treated at the same concentration (5 μg/ml). The induction of HBD-2 mRNA was not significantly different between the tested strains. Values are given as mean ± standard deviation. N = 3. *: p < 0.05.

**Figure 2:**
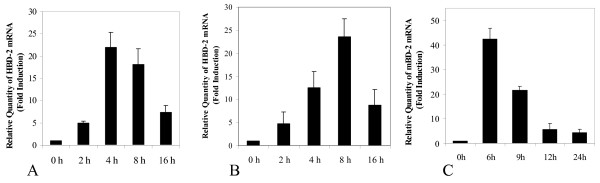
**NTHi whole cell lysate (WCL) up-regulates β-defensin 2 (HBD-2) gene expression in immortalized human mucosal epithelial cells (HMEEC-1).** NTHi treatment of HMEEC-1 (A) and A549 epithelial cells (B) increased the mRNA levels of β-defensin 2. The x-axis shows the time during which the cells were exposed to NTHi WCL (5 μg/ml). The y-axis represents the signal derived from quantitative PCR on total RNA extracted from the cells. (C), NTHi also up-regulates mouse β-defensin 2 (mBD-2) mRNA expression in the mouse middle ear, *in vivo*. NTHi WCL (10 μl) was transtympanically inoculated into the middle ears of anesthetized C57BL/6J mice. Animals were sacrificed at the indicated time points and total RNA extracted from the middle ear mucosa (n = 3). Values are given as mean ± standard deviation.

To find out whether NTHi can increase expression of HBD-2 mRNA *in vitro*, we first treated human middle ear epithelial cells (HMEEC-1) with various NTHi preparations – live NTHi, WCL, pellet, and lipooligosaccharide (LOS). The greatest induction of HBD-2 expression was observed with the NTHi WCL preparation, which suggests that the ligand(s) may be a soluble protein. Even though the other preparations also induced HBD-2, the levels were negligible compared to that of WCL (Fig 1A). Similar experiments using WCL preparations from different strains of NTHi showed no significant differences between the various NTHi strains in inducing HBD-2 expression (Fig 1B). Fig 2A shows the kinetics of HBD-2 expression upon exposure to 5 μg/ml of NTHi WCL. As is evident from the figure, the expression peaked at 4 hours post-treatment then gradually waned out. The induction of HBD-2 by NTHi was also observed in A549 cells (a human lung carcinoma cell line), with near-maximal levels of expression occurring at 8 hours post-treatment (Fig 2B).

#### In vivo

Mice middle ear epithelium inoculated with 10 μl of NTHi WCL showed very similar kinetics to the *in vitro *cell line data above (Fig 2C).

### The TLR2-depedent MyD88-IRAK-TRAF6 signaling cascade is involved in NTHi-induced up-regulation of β-defensin 2 mRNA

#### Antibody blocking and knock-out animal experiment

**Figure 3:**
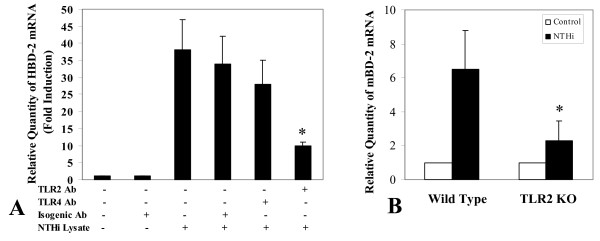
**The TLR2 blocking antibody inhibition of NTHi induced HBD-2 mRNA expression in HMEEC-1 cells and in TLR2 knock out mice.** (A) Pretreatment of HMEEC-1 with human TLR2 blocking antibody for 30 minutes (10 μg/ml, clone TL2.1, eBioscience) reduced the HBD-2 mRNA expression dramatically in response to NTHi lysate stimulation (5 μg/ml). In contrast, human TLR4 blocking antibody did not show any significant effect suggesting that TLR2 is one of the major signaling receptors for inducing human β-defensin 2 expression in response to NTHi stimulation in HMEEC-1. The control group was treated with an isogenic antibody. (B) NTHi WCL-induced expression of murine β-defensin 2 mRNA (mBD-2) in wild type (C57 BL/6) and TLR2^-/- ^mice was compared. Mice were injected with 10 μl of NTHi WCL transtympanically in the left ear. The right ear served as a PBS injected control. Four hours post-injection, the total RNA was harvested and Defb2 expression measured by quantitative PCR (n = 3). Values are given as mean ± standard deviation. N = 3. *: p < 0.05.

Having demonstrated that NTHi can up-regulate β-defensin 2 mRNA in HMEEC-1, we next sought to identify the cell surface receptor that is responsible for recognizing NTHi ligand(s) and initiating the appropriate signaling cascade. Pre-incubation of HMEEC-1 with a TLR2 specific blocking antibody dramatically reduced the expression of HBD-2 in HMEEC-1 cell culture in response to NTHi WCL treatment (Fig 3A), indicating that TLR2 plays a major role in this pathway. The importance of TLR2 in NTHi WCL-induced β-defensin 2 production was also tested in gene knock out animals. The induction rate of mBD-2 expression in TLR2 KO mice middle ear epithelial cells in response to NTHi WCL was substantially lower than that of wild type mice (Fig 3B). This *in vivo *experiment confirmed the critical role of TLR2 in NTHi induced cell signaling that resulted in the production of the highly potent antimicrobial innate immune molecule – β-defensin 2 – by the middle ear epithelial cells.

#### siRNA

**Figure 4:**
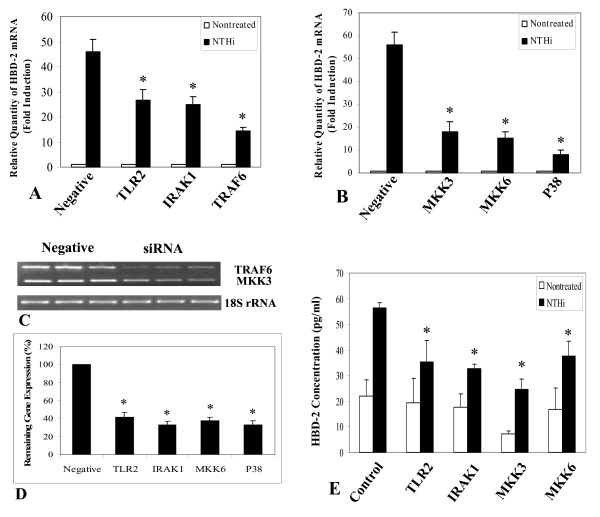
**NTHi-induced HBD-2 expression in HMEEC-1 utilizes the TLR2-dependent IRAK-TRAF6-p38MAPK signaling cascade.** (A and B) Cells treated with siRNA specific for TLR2, interleukin receptor associated kinase 1 (IRAK1), tumor necrosis factor receptor associated factor 6 (TRAF6), MAP kinase kinase 3 (MKK3), MKK6 and p38 showed significantly lower HBD-2 expression compared to a negative control. (C) The targeted gene knock down using siRNA was confirmed either by RT-PCR or(D) quantitative PCR. (E) Reduced HBD-2 peptide production in the HMEEC-1 cells treated with target specific siRNA, after treatment with NTHi lysate. The siRNA knock down cells were treated with NTHi lysate for 24 hours. The culture supernatant was collected and HBD-2 protein levels were measured by ELISA. The HBD-2 secretion from cells with target gene knock down was substantially lower than in negative siRNA treated controls. In experiments A, B, D and E, values are given as mean ± standard deviation. N = 3. *: p < 0.05.

We also used TLR2 knock-down experiments to assess the involvement of TLR2 in the pathway using small interfering RNA (siRNA) specific for TLR2. As shown in Fig 4A, HBD-2 induction by NTHi was inhibited in the presence of TLR2-specific siRNAs. Furthermore, similar siRNA experiments also revealed the involvements of interleukin-1 receptor-associated kinase 1 (IRAK1) and tumor necrosis factor receptor-associated factor 6 (TRAF6) (Fig 4A) as well as MKK3/6 and p38 MAPK (Fig 4B) in NTHi induced β-defensin expression. The efficiency of gene knock down was confirmed by either RT-PCR (Fig 4C) or quantitative PCR (Fig 4D). The Q-PCR results showed 60–70% reduction of target gene expression in TLR2, IRAK1, MKK6 and p38 siRNA treated HMEEC-1 compared with those of negative control siRNA treated cells (Fig 4D). In the presence of 5 μg/ml of NTHi WCL for 24 hours, β-defensin 2 production was also inhibited when cells were transfected with TLR2, IRAK1, MKK3 and MKK6 siRNAs, indicating the direct involvement of these proteins in mediating the NTHi-induced HBD-2 expression (Fig 4E).

#### Dominant negative (DN) plasmids

**Figure 5:**
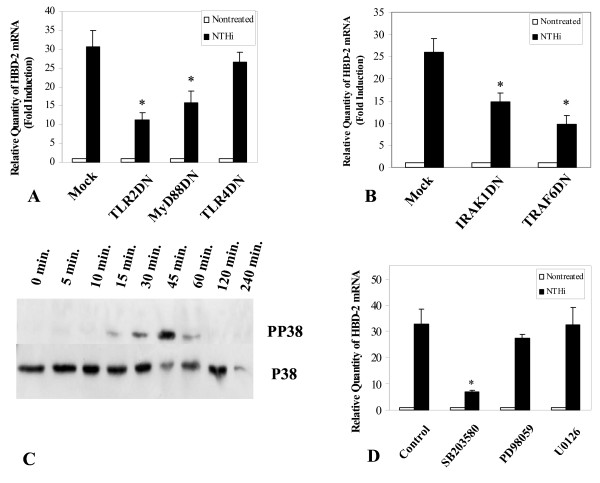
**(A) TLR2 and MyD88 dominant-negative (DN) mutants, but not TLR4 DN mutant, inhibit NTHi-induced up-regulation of HBD-2 expression.** (B) The MyD88 downstream signaling proteins, IRAK1 and TRAF6 are also involved in this up-regulation. (C) The kinetics of P38 phosphorylation: phosphorylation of p38 MAPK started at 15 minutes post-treatment, peaked at 45 minutes post-treatment and then disappeared after 60 minutes. (D) Inhibition of p38 MAPK by SB203580, a specific inhibitor of p38, abolishes HBD-2 up-regulation by NTHi. Cells were pretreated with 5 μM of SB203580, PD98059, and U0126 for 1 hour and then treated with NTHi WCL (5 μg/ml) for 4 hours. Only SB203580 exhibited inhibitory activity towards NTHi WCL-specific HBD-2 expression. The ERK inhibitors, PD98059 and U0126 showed little or no effect. In experiments A, B and D, values are given as mean ± standard deviation. N = 3. *: p < 0.05.

To validate the involvement of MyD88, IRAK1, and TRAF6 in the pathway, we over-expressed dominant-negative mutant (DN) plasmids of each gene associated with the pathway. TLR2 DN, but not TLR4 DN, resulted in inhibition of HBD-2 expression, confirming that TLR2 is the main receptor that mediates NTHi signaling through induction of β-defensin 2 (Fig 5A). The adaptor molecule MyD88 has previously been shown [13,35] to be the first molecule in the signaling cascade, immediately downstream of TLR2. Cells treated with MyD88DN show a substantial reduction in HBD-2 expression (Fig 5A), validating the involvement of this molecule in the pathway. The reduction of HBD-2 expression in IRAK1 and TRAF6 dominant-negative mutants over-expressed cells confirmed the involvement of these genes in the NTHi induced up-regulation of HBD-2 (Fig 5B).

### The p38 MAP Kinase pathway is involved in the up-regulation of β-defensin 2 by NTHi

As shown in Fig 5C, treatment of HMEEC-1 with NTHi WCL (5 μg/ml) results in the rapid phosphorylation of p38 MAPK protein. The phosphorylation peaked at 45 minutes post-treatment and rapidly disappeared 60 minutes after treatment. Exposure to SB203580, a specific inhibitor of p38 α and β, exhibited an inhibition in β-defensin 2 expression, while treatment with PD98059 and U0126, both inhibitors of MEK, did not show any significant effects (Fig 5D).

## Discussion

Antimicrobial innate immune molecules produced by the epithelial cells provide the host with a constitutive or immediately inducible defense mechanism against invading pathogens. Under normal conditions, the middle ear of humans and laboratory animals remains sterile, although OM pathogens, which are part of the normal nasopharyngeal flora can reach the middle ear via the Eustachian tube causing infection [45]. Furthermore, non-inflamed tubal and middle ear mucosa have been shown to contain relatively few immunocytes [46,47]. All of these findings suggest that the components of the innate immune system may be important in the defense of the tubotympanum (middle ear and Eustachian tube). Furthermore, due to the premature and under-developed adaptive immune system in infants and young children, the role of AIIMs in protecting against OM pathogens becomes of paramount importance. Park and Lim [10] showed that the lysozyme positive cells in the epithelium and glandular structures of the Eustachian tube of adult and early post-natal animals were similar. Our previous studies also showed synergistic antimicrobial activities of AIIMs against OM pathogens [8,14]. These results lend strong support to the hypothesis that AIIMs protect the tubotypmpanum during the neonatal and early postnatal periods when the adaptive immunity is not yet fully developed.

Among the AIIMs, β-defensin 2 plays a pivotal role against major OM pathogens and previous studies have shown that this molecule is inducible by a variety of agents, including cytokines, NTHi and its components/molecules [8,14,23,27,30]. Furthermore, we have previously shown that this molecule is expressed at high levels in the middle ear mucosa of patients with OM, but it is undetectable in the normal middle ear mucosa [27].

Some LOS mutants of NTHi exhibit reduced virulence and require higher doses in order to induce OM [48]. Mutations in certain bacterial membrane components alter the susceptibility of the mutants towards AIIMs [49,50]. However, these mutations did not significantly affect the inducibility of HBD-2 expression. To date, the exact nature and identity of the β-defensin 2 inducing molecule(s) remains elusive, but what is known is that it is maximally active and is present in the soluble WCL portion (Fig 1A and 1B) [51]. Currently several NTHi macromolecules have been identified as vaccine candidates, which include adhesins, high molecular weight adhesins, pilus proteins, outer membrane proteins and LOS [52-55]. Identification of induction of the responsible ligand for beta-defensin 2 requires more research.

Treatment with NTHi WCL causes a substantial increase in HBD-2 mRNA expression in both HMEEC-1 and A549 cells. However, the kinetics of HBD-2 expression is a little different between the two cell lines (Fig 2A and 2B), which may be the result of the fact that different cells respond differently even to the same bacterial components [20]. *In vivo *induction of mouse β defensin-2 with NTHi WCL treated mice middle ear epithelia exhibits a similar trend to that of HMEEC-1; the mRNA level peaked at 6 hour post-inoculation and dropped back down to basal levels by 12 hours (Fig 2C). This rapid increase in mouse β defensin-2 levels may indicate that the middle ear epithelial cells, but not inflammatory cells, were the sole source of the mouse β defensin-2 mRNA – β defensins are mainly produced by epithelial cells since monocytes would not have yet begun infiltrating the middle ear cavity during this period [56].

The Toll-like receptors (TLRs) have been shown to be critical for the recognition of bacteria and recent studies have demonstrated that TLR2 and TLR4 are involved in NTHi signaling [3,57]. To test the involvement of either TLR2 or TLR4 in mediating NTHi WCL induced HBD-2 up-regulation, we used blocking antibodies, siRNA, and dominant negative mutants of TLR2 and TLR4 to inhibit the receptors' activities (Figs 3 – 5). All three methods revealed TLR2 as being the primary receptor for the recognition of NTHi WCL component.

Based on these results, we investigated the role and identity of other possible players downstream of the TLR2 pathway that may be involved in HBD-2 up-regulation by the NTHi WCL such as MyD88, which was previously shown to be the first molecule in the signaling cascade immediately downstream of the TLR2 [13] and its associated proteins, interleukin-1 receptor-associated kinase 1 (IRAK1) [13,19] and tumor necrosis factor receptor-associated factor 6 (TRAF6). MyD88 is recruited via its TIR death domain and promotes association and phosphorylation of the IRAK1. The phosphorylation of IRAK1 results in its dissociation from the complex and its interaction with TRAF6. This interaction results in the activation of downstream signaling in the majority of the epithelial cells studied [13,19]. Our results using siRNA (Fig 4A, 4B &4E) and DN plasmids (Fig 5A &5B) indicate that MyD88, IRAK1 & TRAF6 are directly involved in regulating HBD-2 expression in the presence of NTHi WCL.

Activation of the TLR2-MyD88-IRAK1-TRAF6 can lead to the activation of many downstream signaling pathways, including members of the mitogen-activated protein kinase (MAPK) family [19,35]. Previous studies have suggested that the p38 MAPK pathway is involved in HBD-2 induction in other cell systems [20,58,59]. We therefore sought to determine if p38 MAPK can act as a major signaling mediator for NTHi induced β-defensin 2 expression. Indeed, our results support data from previous work since such up-regulation was greatly inhibited by SB203580, a specific inhibitor of p38 MAPK but not by ERK inhibitors such as PD98059 and U0126. Also we used siRNA gene knock down to confirm the involvement of p38 MAPK and its associated upstream activators – MKK3 and MKK6. The results (Fig 4B &4E) support our hypothesis that NTHi WCL-specific HBD-2 up-regulation in HMEEC-1 takes place through the p38 MAPK cascade. The kinetics of this signaling was very rapid; phosphorylation of p38 MAPK started 15 minutes after stimulation and peaked at 45 minutes (Fig 5C). Near complete dephosphorylation was achieved by 60 minutes post-stimulation. Secretion of the β-defensin 2 was also reduced in cells treated with siRNAs targeted for down-regulating TLR2, IRAK1, TRAF6, and MKK(3/6) signaling molecules compared to a negative control. The importance of the role of TLR2 in NTHi WCL induced β-defensin 2 expression was also confirmed *in vivo*. The induction of mouse β-defensin 2 (mBD-2) mRNA by NTHi WCL in TLR2 KO mice middle ear was much lower than that of an age matched wild type control.

Beta-defensin exhibits not only innate immune activity such as direct killing of invading bacteria but also has been known to mediate adaptive immunity [60]. The secreted β-defensin functions as an chemoattractant for CCR6 positive immature dendritic and memory T cells, facilitating the transition from innate immunity to adaptive immunity by producing inflammatory cytokines and chemokines such as IL-1 [19,60-62]. Our previous studies showed that IL-1α is a potent inducer of β-defensin 2 and acts synergistically with NTHi WCL to up-regulate β-defensin 2 expression [27,30]. These experiments showed that IL-1α-induced β-defensin 2 expression was mediated through the Src-dependent Raf-MEK1/2-ERK MAPK pathway and uses the same TIR domain of TLR2 that recognizes the unknown NTHi WCL component [27,34]. The synergism between IL-1α and NTHi may be explained as follows: NTHi WCL stimulates HMEEC-1 through MyD88 dependent TLR2 signaling pathway inducing an early phase response with subsequent β-defensin 2 and proinflammatory cytokine (such as IL-1α) production [13,34]. The secreted IL-1α may further stimulate the cells through MyD88 independent IL-1R pathway that initiates a late phase response [13,34]. Therefore, even though active NTHi component/ligand and IL-1α utilize the same TIR domain and are working through the p38 and ERK MAPK signaling pathways respectively, they can act synergistically to amplify β-defensin 2 expression [13,34,63].

Recently a new group of receptors, Nacht-LRR, NOD-like Receptor, or CATERPILLAR (NLR), has been reported that resemble and function as intracellular receptors for bacterial recognition [19,64,65]. These receptors not only recognize invasive microbes and appropriate bacterial components/ligands, but also play pivotal roles in triggering inflammatory response by converting the inactive forms of pro-cytokines to active mature cytokines [66,67]. Based on these facts, we speculate that these intracellular receptors may contribute to increasing the NTHi induced β-defensin 2 expression in HMEEC-1. Although NTHi is considered as a mucosal surface pathogen, there have been reports that NTHi can be internalized in the mucosal epithelium [68,69]. While the significance of this bacterial internalization in the pathogenesis of otitis media is not yet known, it is possible that such internalized bacterial components can also induce β-defensin up-regulation. Studies are currently underway in our laboratory to test this possibility.

## Conclusion

In conclusion, we have observed that NTHi WCL signals through a TLR2 dependent TIR domain similar to IL-1α. However, this is where the similarities end: Both NTHi WCL and IL-1α signal through entirely different sets of downstream players, possibly leading to a synergistic induction of HBD-2 in infection.

## Competing interests

The authors declare that they have no competing interests.

## Authors' contributions

H–YL performed most of experiments and data analysis. TT performed the cell culture and DN transfection study. JS performed the siRNA study. AA performed the antibody blocking and Q-PCR analysis. J–IW participated in the siRNA study and helped Q-PCR. HP performed the western blot and ELISA studies. SKM prepared the lysate of NTHi, and assisted experiments related with siRNA. AA participated in study design and data analysis. RKP extracted RNA and performed Q-PCR study. S–HK participated in cell culture and Q-PCR analysis. S–SK collected mouse middle ear specimens and helped with Q-PCR. RG participated in data analysis and helped to draft the manuscript. DJL is recipient of NIDCD R01 DC05025-04, which supported this work and the studies were conducted in his laboratory, with him as the principal investigator. All authors read and approved the final version of the manuscript.

## Pre-publication history

The pre-publication history for this paper can be accessed here:


